# Highly Efficient Photocatalytic Degradation of Tetracycline Antibiotics by BiPO_4_/g-C_3_N_4_: A Novel Heterojunction Nanocomposite with Nanorod/Stacked-like Nanosheets Structure

**DOI:** 10.3390/molecules30142905

**Published:** 2025-07-09

**Authors:** Xin Zhu, Moye Luo, Cheng Sun, Jinlin Jiang, Yang Guo

**Affiliations:** 1 State Environmental Protection Key Laboratory of Soil Environmental Management and Pollution Control, Nanjing Institute of Environmental Sciences, Ministry of Ecology and Environment of China, Nanjing 210042, China; zhuxin@nies.org (X.Z.);; 2 State Key Laboratory of Pollution Control and Resource Reuse, School of the Environment, Nanjing University, Nanjing 210023, China

**Keywords:** heterojunction photocatalysts, BiPO_4_/g-C_3_N_4_, photodegradation mechanism, tetracyclines

## Abstract

The use of semiconductors for photocatalytic degradation of organic pollutants has garnered considerable attention as a promising solution to environmental challenges. Compared to TiO_2_, BiPO_4_ exhibits superior photocatalytic activity. However, its large band gap restricts its light absorption to the UV region. One effective technique for extending BiPO_4_’s absorption wavelength into the visible spectrum is the construction of the heterostructure. This study aimed to synthesize monodisperse BiPO_4_ nanorods via a solvothermal approach and fabricate BiPO_4_/g-C_3_N_4_ heterojunctions with varying loadings through in situ deposition. Tetracyclines were employed as the target pollutant to evaluate the photocatalytic performance and stability of the prepared materials. The results indicated that 5 wt% of composite exhibited better photocatalytic performance than single catalysts, which showed the highest photodegradation efficiency of approximately 98% for tetracyclines. The prepared bi-photocatalyst presented favorable stability under sunlight irradiation, the photocatalytic activity of which remained almost unchanged after four cycles. The enhanced photocatalytic activity was attributed to the synergistic effect. Additionally, the possible degradation mechanism was elucidated utilizing the semiconductor energy band theory. Overall, this work presents new perspectives on synthesizing innovative and efficient visible-light-driven photocatalysts. It also offers a mechanistic analysis approach by integrating theoretical calculations with experimental observations.

## 1. Introduction

With the rapid advancement of science and technology, the escalating use of antibiotics in medical treatment, livestock, and aquaculture has led to their widespread detection in natural water bodies and other environmental media [1,2,3]. Tetracycline (TC), as a basic broad-spectrum bacteriostatic agent among the most commonly used tetracycline family of antibiotics in the breeding industry, has been extensively applied worldwide [4]. TC boasts strong water solubility, a long half-life, and structural stability under acidic conditions, which can persist for a long time in natural water bodies and soils while participating in the material circulation process [5]. However, a variety of antibiotic resistance bacteria may be induced by the residual TC in the environment, affecting the normal life activities of native organisms and potentially threatening humans and the ecosystem through the transmission and enrichment of the food chain [6]. In response, extensive research and efforts have been devoted to the rapid degradation of residual tetracycline antibiotics in the environment.

Initially, TC in wastewater is expected to be removed via conventional sewage treatment processes such as physical adsorption and biological treatment. However, the removal efficiency is severely hindered by the complex structure and stable physicochemical properties of TC antibiotics. Additionally, this challenge is compounded by potential risks of secondary pollution [7,8]. In recent years, photocatalytic oxidation technology—characterized by its green chemistry principles, high efficiency, and absence of secondary pollution—has emerged as a promising advanced treatment for TC from wastewater, attracting significance research attention. Muller et al. [9] and Fujishima et al. [10] discovered that the photocatalytic oxidation process had the ability to efficiently destroy the structure of organic pollutants in wastewater and to achieve removal of pollutants in the 1970s. Since then, the photocatalytic oxidation process has been extensively studied in the field of antibiotic pollution treatment in wastewater, the main research fields of which focus on optimizing the photocatalytic degradation process and synthesizing innovative and efficient photocatalysts. Chen Yan et al. [11] prepared N-doped TiO_2_/diatomite-integrated photocatalytic particles (N-IPP), which achieved approximately 85% tetracycline removal efficiency after 5 reuse cycles. Recently, Tang Tao et al. [12] reported a new type of p-n heterojunction photocatalyst Bi_2_O_3_/Ti^3+^-TiO_2_. The photocatalytic degradation efficiency of tetracyclines under visible light irradiation was enhanced by 15% compared with traditional TiO_2_. However, traditional TiO_2_-based photocatalysts still faced several limitations, including low utilization efficiency of sunlight, low photocatalytic quantum efficiency, and insufficient photocatalytic reaction driving force.

Extensive research has explored the photocatalytic potential of various novel semiconductor materials to develop alternatives for TiO_2_ with superior photocatalytic performance [13,14,15,16]. Due to their characteristics of non-toxic properties, strong physicochemical stability, enhanced visible light response, and suitable energy bandwidth, bismuth-based semiconductors have attracted extensive attention as novel semiconductor photocatalysts for the degradation of antibiotics [4,17,18,19,20]. As initially reported by Pan’s research team [21],bismuth phosphate (BiPO_4_) showed excellent performance in degrading organic pollutants as a photocatalyst. This efficacy was primarily attributed to the efficient electron–hole separation due to the inherent inductive effect of the highly negatively charged PO_4_^3−^ groups [22,23]. However, the band bap of BiPO_4_ was appropriately 3.85 eV, and the relatively high band gap resulted in the limitation of the light absorption range to the short-wavelength ultraviolet region. Concurrently, the inherent structural defect severely limited its photocatalytic efficiency under visible light.

Heterojunction catalysts constructed by doping non-metallic semiconductors in bismuth-based materials generally exhibit higher photocatalytic efficiency over single semiconductors. The energy level difference between the compound semiconductors would aid the separation of photogenerated electron pairs and reduce their recombination efficiency, thereby enhancing the photocatalytic activity of heterojunction photocatalysts [24,25]. Graphite-like carbon nitride (g-C_3_N_4_) was considered as highly promising for enhancing catalytic degradation performance of metal–semiconductor photocatalysts toward tetracycline, owing to its high chemical and thermal stability, non-toxicity, and strong visible-light catalytic activity even at non-nanoscale [26]. Hyung Jun Kong et al. [27] prepared a composite photocatalyst with bismuth vanadate (BiVO_4_) and sulfur-doped g-C_3_N_4_ for water oxidation. Under identical reaction conditions, the oxygen release rate was demonstrated to be twice that of BiVO_4_, while its photon efficiency also increased by 19%. Jielin Yuan et al. [28] synthesized a nanoscale phosphorus-doped g-C_3_N_4_ and BiPO_4_ composite catalyst with a hydrolysis rate of up to 1110 μmol h^−1^ g^−1^. However, limited research has been conducted on the enhancement effect of heterojunction photocatalyst BiPO_4_/g-C_3_N_4_ on the photocatalytic oxidation of tetracyclines in wastewater. Additionally, the specific contributions and underlying mechanisms driving the improvement in photocatalytic performance remain underexplored.

In this study, the BiPO_4_/g-C_3_N_4_ heterojunction photocatalysts with varying loadings were synthesized using the in situ precipitation method. The morphology, structure, and photoelectrochemical properties were characterized, and the photocatalytic performance and stability of the prepared heterojunction catalysts for tetracyclines were investigated. In addition, the experimental observations were integrated with theoretical calculations to further delve into the mechanism of the formation of the heterojunction and the effective separation of the charge carriers at the heterojunction interface.

## 2. Results and Discussion

### 2.1. Crystal Structure and Morphology

**Figure 1:**
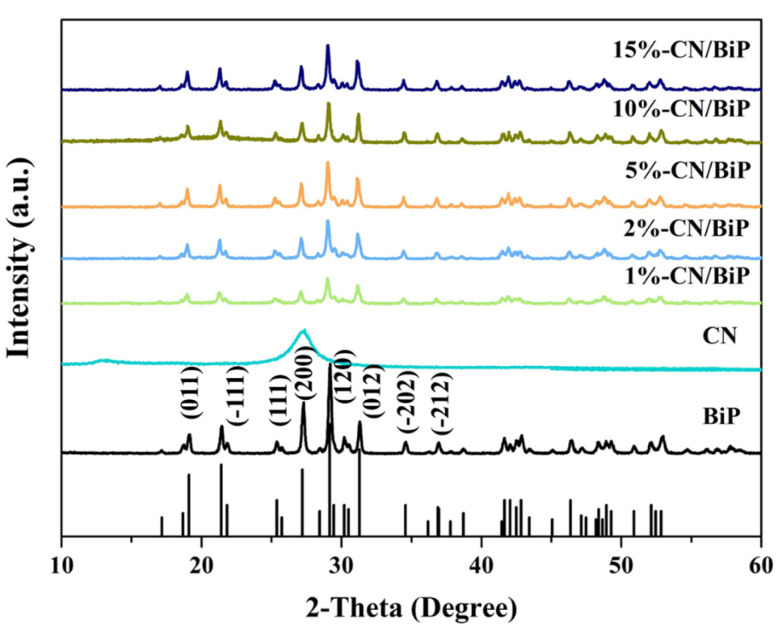
XRD spectra of BiPO_4_, g-C_3_N_4_, and composite CN/BiP with varying mass ratios.

The crystal structures and purity of BiPO_4_, g-C_3_N_4_, and composite g-C_3_N_4/_BiPO_4_ (CN/BiP) were analyzed by X-ray diffraction (Figure 1). The characteristic peaks of g-C_3_N_4_ at 13.0° and 27.4° corresponded to the crystalline planes of repetitive structure (100) and the crystalline planes of lamellar stacking (002) in the same plane of g-C_3_N_4_, respectively (JCPDS-50-0367). The characteristic peak at 13° was caused by the in-plane structural stacking of the aromatic ring system, corresponding to a lattice spacing of 0.679 nm. The strongest peak at 27.4° was the typical interlayer stacking peak of the aromatic ring system, with the lattice spacing value reflecting the intercalation spacing. For the prepared BiPO_4_, the peaks at 19.0°, 21.3°, 25.3°, 27.2°, 29.1°, 31.2°, 34.5°, and 36.9° corresponded to the (011), (-111), (111), (200), (120), (012), (-202), and (-212) crystal planes, respectively (JCPDS 80-0209). With the increasing content of g-C_3_N_4_, diffraction peaks corresponding to BiPO_4_ were detectable in all the CN/BiP samples, indicating that the introduction of g-C_3_N_4_ did not alter the crystal structure of BiPO_4_. However, the existence of g-C_3_N_4_ could not be determined by the XRD across different scales of composite CN/BiP samples due to the close proximity of the BiPO_4_ characteristic peak (200) and the g-C_3_N_4_ characteristic peak (002), with the latter being masked by the former.

**Figure 2:**
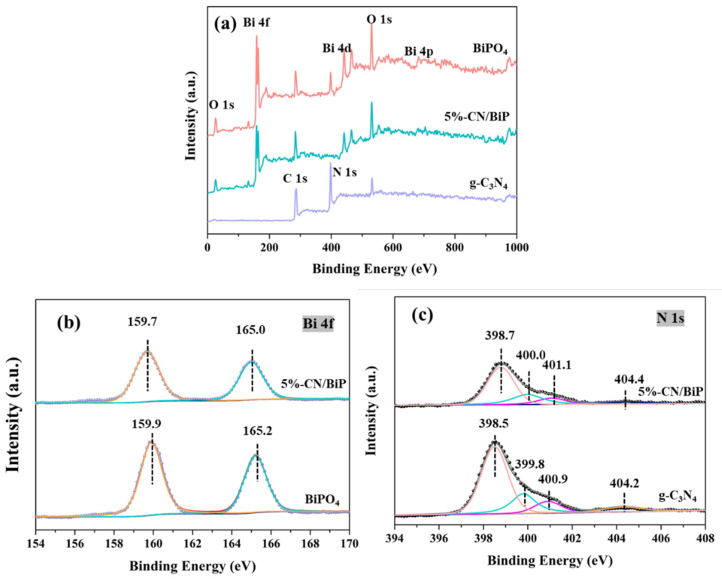
(**a**) XPS survey spectra of BiPO_4_, g-C_3_N_4_ and 5%-CN/BiP; High-resolution XPS spectra, (**b**) Bi 4f of BiPO_4_ and 5%-CN/BiP, and (**c**) N1s of g-C_3_N_4_ and 5%-CN/BiP.

The X-ray photoelectron spectroscopy data revealed the surface elemental composition and chemical valence states of BiPO_4_, g-C_3_N_4_, and composite CN/BiP (Figure 2). The XPS spectra clearly showed the coexistence of C, N, Bi, P, and O elements in 5%-CN/BiP, confirming the successful synthesis of CN/BiP composites (Figure 2a). The high-resolution XPS spectra of Bi 4f uncovered two characteristic peaks Bi 4f_7/2_ and Bi 4f_5/2_ of BiPO_4_ with binding energies of 159.9 eV and 165.2 eV, respectively [29] (Figure 2b). Moreover, a negative migration of 0.2 eV in the binding energy of Bi 4f was observed in the BiP/CN system compared to pure BiPO_4_. The N 1s spectrum of g-C_3_N_4_ was divided into four peaks at a binding energy of 398.5 eV, 399.8 eV, 400.9 eV, and 404.2 eV, respectively (Figure 2c). The characteristic peaks at binding energies of 398.5 eV and 399.8 eV corresponded to pyridine nitrogen (N- sp^2^ C) and pyrrole nitrogen (N- sp^3^ C) for the triazine units (C-N=C), respectively. The latter two were assigned to C-N-H and N-N configurations, consistent with previous literature reports [29]. In contrast, the N 1s peak of the 5%-CN/BiP sample shifted towards the higher binding energy. Generally, a negative correlation existed between the binding energy and the surface electron density [30]. The binding energy shifts of Bi 4f and N 1s in the XPS spectra indicated that the BiPO_4_ and g-C_3_N_4_ were not merely physically mixed but formed a heterojunction between the two semiconductors. Despite both being n-type semiconductors, g-C_3_N_4_ acted as the electron donor in the composite, and electrons were transferred from g-C_3_N_4_ to BiPO_4_ upon heterojunction formation.

Photocatalyst performance is closely related to its particle size and morphological structure [31]. Herein, during the photocatalytic processes, the time for randomly generated photogenerated electron–hole pairs to diffuse from the interior of the photocatalyst to the surface could be obtained from Equation (1) [32].

τ = r^2^/π^2^D (1)

where r is the particle size radius, and D is the diffusion coefficient of the photogenerated carriers. Smaller crystal sizes of photocatalysts enable faster diffusion of charge carriers to the interface, where they react with free radicals or target pollutants.

**Figure 3:**
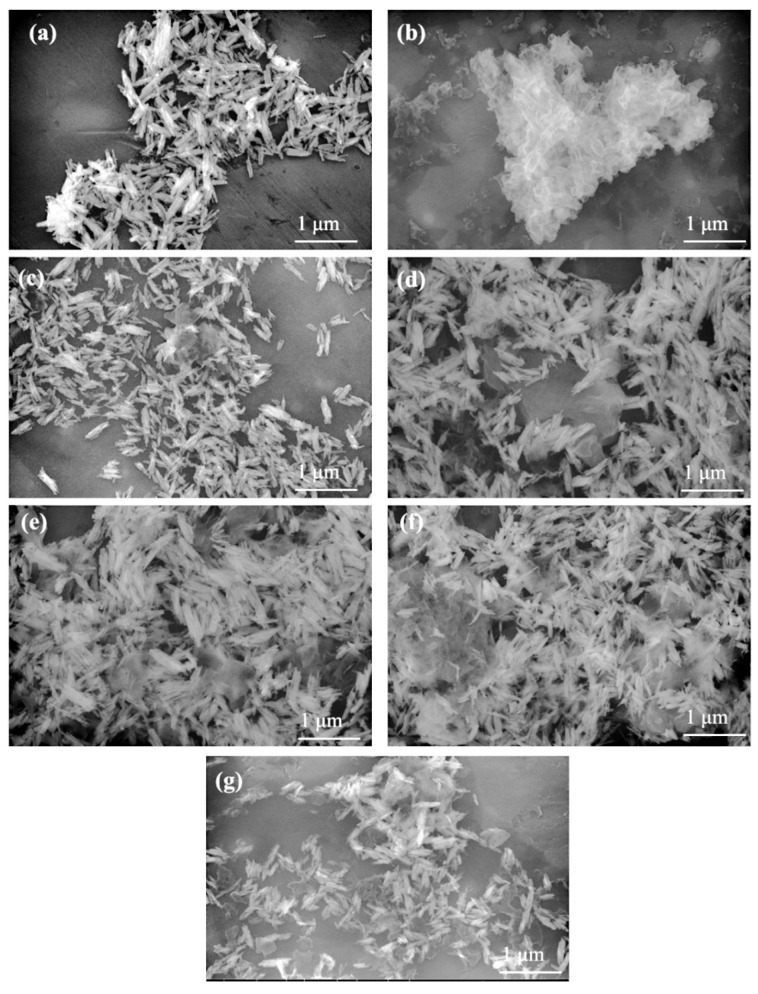
SEM images for (**a**) BiPO_4_, (**b**) g-C_3_N_4_, (**c**) 1%-CN/BiP, (**d**) 2%-CN/BiP, (**e**) 5%-CN/BiP, (**f**) 10%-CN/BiP, and (**g**) 15%-CN/BiP.

SEM images of BiPO_4_ indicated that the BiPO_4_ nanorods synthesized in this study using the solvothermal method featured a smooth surface and homogeneous morphology with an average diameter and length of approximately 50–70 nm and 600 nm, respectively (Figure 3a). One-dimensional (1D) nanostructured materials (e.g., nanowires, nanorods, nanotubes) facilitated efficient separation of photogenerated electron–hole pairs. This stemmed from the ability of charge carriers to undergo ballistic charge transport along the axial direction [33]. On the other hand, g-C_3_N_4_ was a typical irregularly layered stacking structure, consisting of two-dimensional (2D) lamellae stacked (Figure 3b). The composite of 1D BiPO_4_ and 2D g-C_3_N_4_ flakes would provide more active sites, thereby enabling the diffusion of photogenerated carriers to the interface of the composite and improving the photocatalytic performance [34]. Morphological analyses of CN/BiP materials with varying g-C_3_N_4_ loading ratios revealed that the surface of BiP nanorods exhibited an increasing coverage of g-C_3_N_4_ nanoflakes as the loading of g-C_3_N_4_ increased (Figure 3c–g). When the loading mass ratio of g-C_3_N_4_ exceeded 5%, the nanoflake-like g-C_3_N_4_ tended to agglomerate and accumulate, compromising the dispersion of the samples. Therefore, an optimal loading mass ratio between g-C_3_N_4_ and BiPO_4_ might exist.

**Figure 4:**
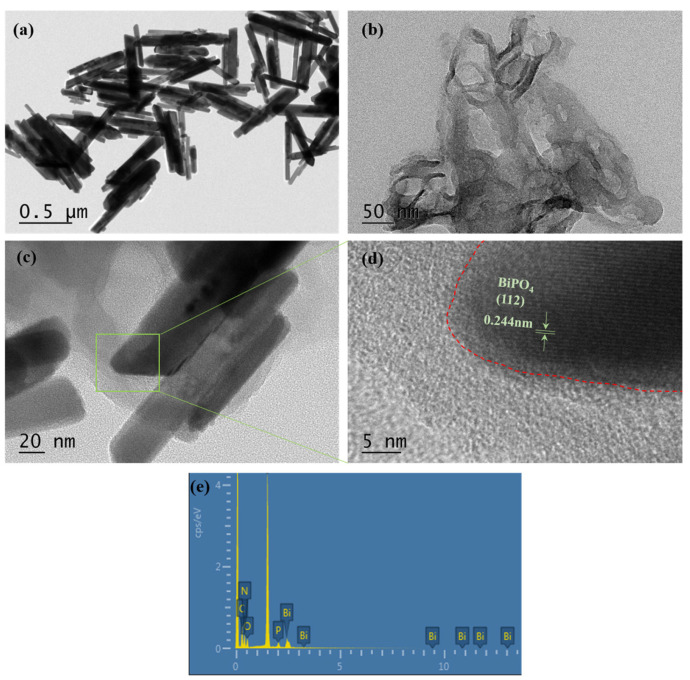
TEM images for (**a**) BiPO_4_, (**b**) g-C_3_N_4_, (**c**) 5%-CN/BiP, (**d**) HRTEM images, and (**e**) EDS line map of the sample 5%-CN/BiP.

TEM and HRTEM characterization of the samples’ microscopic morphology revealed that the structure of BiPO_4_ nanorods and the stacked flake morphology of g-C_3_N_4_ were consistent with the above SEM observations (Figure 4a,b). The microscopic morphology of the samples characterized by TEM showed that the structure of BiPO_4_ nanorods, g-C_3_N_4_ stacked flakes, and the dispersion of BiPO_4_ nanorods in g-C_3_N_4_ flakes aligned with the SEM results (Figure 4a–c). The HRTEM image of the 5%-CN/BiP sample suggested a visible interface region between the two semiconductors, which had been marked with red dotted line. The lattice stripe with a spacing of 0.244 nm corresponded to the (112) crystalline plane of BiPO_4_, while the amorphous region was attributed to g-C_3_N_4_ (Figure 4d). The results were consistent with the XRD data, confirming the successful construction of heterojunctions between the two photocatalysts.

**Figure 5:**
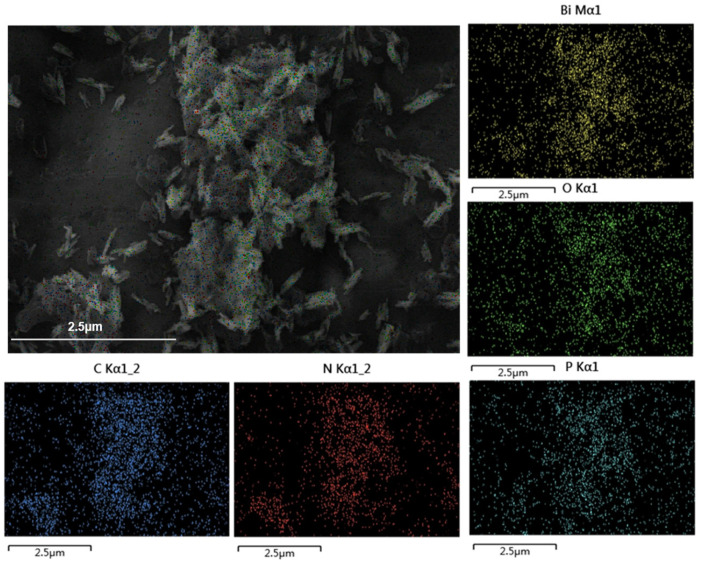
FESEM-EDS mapping of 5%-CN/BiP.

The FESEM-EDS analysis showed that the synthesized 5%-CN/BiP sample contained five elements, including Bi, P, O, C, and N (Figure 4e). These elements were uniformly distributed in the composite catalyst, further proving the successful synthesis of the CN/BiP sample (Figure 5).

### 2.2. Photoelectrochemical Properties

**Figure 6:**
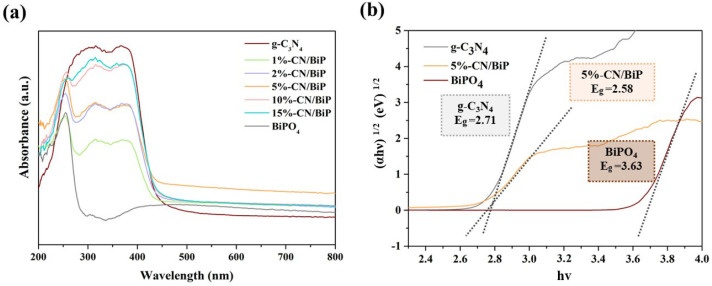
(**a**) DRS spectra of single BiPO_4_, g-C_3_N_4_, and composite CN/BiP with different mass ratios; and (**b**) (αh*v*)^2^/_(_αh*v*)^1/2^versus photon energy (h*v*) of the as prepared BiPO_4_, g-C_3_N_4_, and 5%-CN/BiP.

The photoelectrochemical properties of the samples were characterized using DRS, PL, photocurrent, and EIS impedance. The optical absorption properties of single BiPO_4_ samples and CN/BiP samples with varying mass ratios were analyzed via DRS. The results indicated that BiPO_4_ could only respond to UV light, with the absorption band edge at roughly 300 nm (Figure 6a). The absorption spectra of the CN/BiP composites with different mass ratios extended from the short UV wavelength to the visible region with absorption edges close to 450 nm. The addition of g-C_3_N_4_ not only expanded the light absorption range of the catalyst to the visible region, but also improved its light response in the absorption region of 260–450 nm. This indicated that the CN/BiP composite had high visible light utilization. The 5%-CN/BiP exhibited the best optical absorption performance in the visible region. The energy bands of the semiconductor were calculated according to the Kubelka–Munk function Equation (2)

αhν = A(hν − E_g_)^n/2^ (2)

where α, ν, A, and E_g_ are the optical absorption coefficient, optical frequency, constant, and forbidden band width, respectively. The value of n is 1 when the bandgap type of the semiconductor is direct bandgap (BiPO_4_) and 4 for indirect bandgap (g-C_3_N_4_) [35]. Herein, the forbidden band widths of the BiPO_4_, g-C_3_N_4_ and 5%-CN/BiP samples were 3.63 eV, 2.71 eV, and 2.58 eV, respectively (Figure 6b). This indicated that the CN/BiP composites exhibited lower band gap energy compared to the single-component samples, a characteristic favorable for the generation of photogenerated electron–hole pairs.

**Figure 7:**
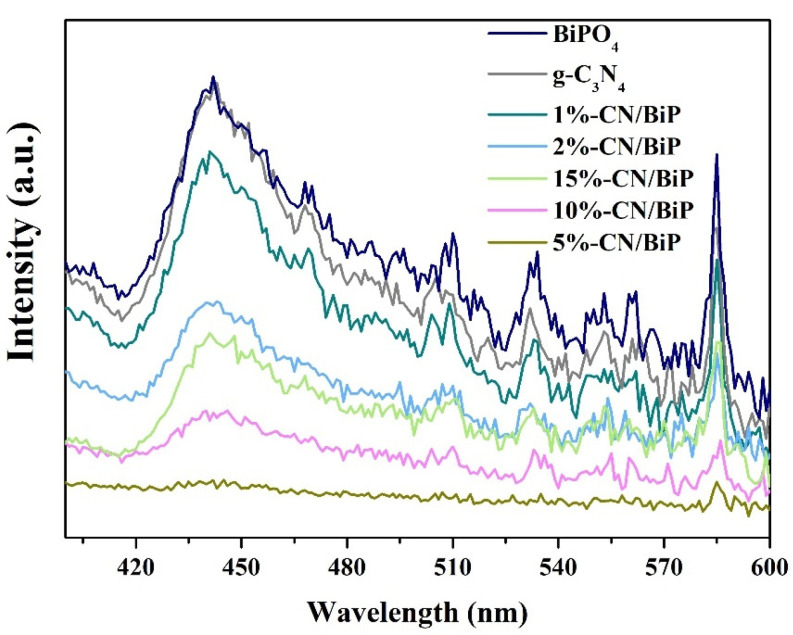
Fluorescence emission spectra of BiPO_4_ and composite CN/BiP with different mass ratios.

The radiative complexation of photogenerated carriers significantly influences the activity of photocatalysts. Fluorescence emission spectra (PL) are frequently employed to investigate the luminescence properties of synthetic materials. Generally, lower PL intensity indicates reduced recombination efficiency of photogenerated carriers, implying higher separation efficiency and superior catalytic performance of the material. Herein, the BiPO_4_ sample demonstrated a strong emission peak at 440 nm with higher intensity compared to all the materials compounded with g-C_3_N_4_ (Figure 7). This indicated that the addition of g-C_3_N_4_ effectively promoted the separation of carriers while reducing the complexation rate, thus improving the activity of the photocatalyst. Under the optimal conditions (5% mass ratio), the photoluminescence intensity was substantially reduced, and the photocatalytic performance was improved, consistent with the DRS results.

**Figure 8:**
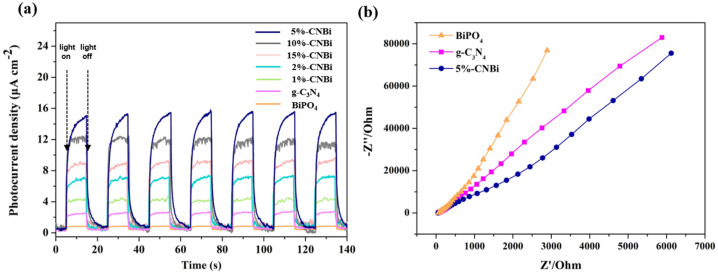
(**a**) Transient photocurrent response; and (**b**) electrochemical impedance spectroscopy of BiPO_4_, g-C_3_N_4_, and CN/BiP composite.

The transient photocurrent responses of the electrodes of BiPO_4_, g-C_3_N_4_, and CN/BiP materials were tested to visually elucidate the separation and migration properties of photogenerated electron–hole pairs on the synthesized materials. Figure 8a presents the electrochemical impedance diagrams (EIS) of the corresponding samples. The results indicated that the photocurrent density of all samples remained stable and electron regenerative after seven light–dark intermittent cycles of experiments. The CN/BiP composites showed higher photocurrent intensity than single BiPO_4_, g-C_3_N_4_ materials, indicating that the composite heterojunctions had more efficient electron–hole separation and migration properties [36]. The smaller electrochemical impedance radius of the 5%-CN/BiP sample implied lower charge transfer resistance and higher charge transfer efficiency, reflecting more effective separation of photogenerated carriers (Figure 8b). The results revealed that the heterojunction of CN/BiP composites was favorable to suppressing the compounding process of electrons and holes, while promoting the migration and separation of charge carriers.

### 2.3. Photocatalytic Degradation Properties

**Figure 9:**
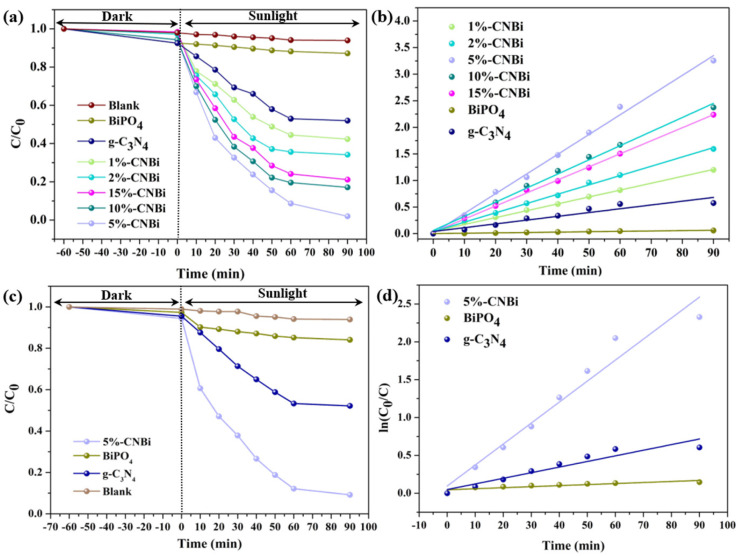
(**a**) Degradation and (**b**) pseudo-first-order kinetics curves of TC over g-C_3_N_4_/BiPO_4_ samples with different molar ratios; as well as (**c**) degradation and (**d**) pseudo-first-order kinetics curves of OTC.

The degradation experiments on TC indicated that the photocatalytic activity of the synthetic catalysts was significantly enhanced compared to the single catalyst. Without the addition of a photocatalyst, the TC concentration remained almost unchanged after 90 min of Xe lamp irradiation, demonstrating its negligible photolytic effect. When BiPO_4_ and g-C_3_N_4_ were employed as photocatalysts, the degradation rates of TC were 12.8% and 48.0%, respectively, after 90 min of irradiation (Figure 9a). The loading of g-C_3_N_4_ had a more pronounced influence on the photocatalytic activity of the composites, among which the 5%-CN/BiP sample demonstrated the optimal photocatalytic activity, with the degradation rate of TC close to 100% at the conclusion of the experiment (Figure 9a).

The photodegradation rate constant of TC could be calculated according to the following quasi-level kinetic Equation (3) of the Langmuir–Hinshelwood model [37]:

ln(C_0_/C) = k_app_t (3)

where the slope k_app_ denotes the apparent rate constant; C is the TC concentration at different reaction times; C_0_ is the initial TC concentration; and t is the time of illumination. The fitted curves of ln(C_0_/C) versus time t exhibited a good linear relationship, indicating that the reaction of sample degradation of TC was in accordance with pseudo-first-order kinetics (Figure 9b). The apparent rate constants of g-C_3_N_4_/BiPO_4_ composites were significantly higher than those of single photocatalysts (Table 1). This enhancement was likely attributed to the formation of a heterojunction between g-C_3_N_4_ and BiPO_4_ in the composite, which substantially improved the material’s photocatalytic performance [38]. In addition, the photocatalytic activity of the synthesized catalyst showed a trend of increasing and then decreasing with the growth of g-C_3_N_4_ loading, and the optimal photocatalytic degradation performance was achieved when the loading of g-C_3_N_4_ was 5 wt%. This phenomenon might arise because excessiveg-C_3_N_4_ wrapped on the surface of the synthesized catalyst restricted the light absorption of the heterojunction and weakened the catalyst’s surface adsorption capacity on TC, thereby reducing the photocatalytic degradation efficiency [36].

Figure 9c,d show the degradation efficiency and kinetics curves of OTC. Similarly to TC, 5%-CN/BiP also demonstrated the best photocatalytic activity. Concurrently, the photodegradation rate constants of BiPO_4_, g-C_3_N_4_ and 5%-CN/BiP samples for OTC were 1.37 × 10^−3^, 7.42 × 10^−3^, and 2.77 × 10^−2^, respectively (Table 1). The above results confirmed that the combination of BiPO_4_ and g-C_3_N_4_ was advantageous to the separation of photogenerated carriers of the photocatalyst, thereby improving the catalytic degradation activity of the sample. Insufficient loading of g-C_3_N_4_ prevented full contact between BiPO_4_ and g-C_3_N_4_, thus hindering heterojunction formation. Excessive loading of g-C_3_N_4_, while ensuring charge transfer, might hinder the absorption of visible light by the material and decelerate the generation rate of photogenerated electrons. Consequently, the 5%-CN/BiP photocatalyst exhibited the best photocatalytic activity, which was also consistent with the previous characterization results.

**Figure 10:**
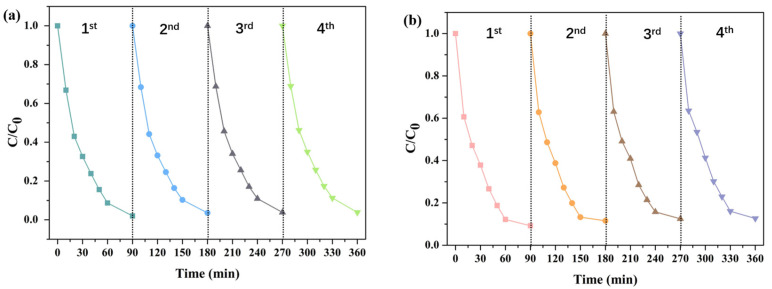
Recycling runs of 5%-CN/BiP for (**a**) TC and (**b**) OTC photodegradation.

From the perspective of practical application, in addition to photocatalytic efficiency, the stability of the photocatalyst was another key factor reflecting its performance. In order to investigate the stability and reusability of 5%-CN/BiP, the cyclic degradation experiments of two tetracycline antibiotics by photocatalysts under sunlight were carried out. As depicted in Figure 10, after four cycles, the photocatalytic degradation efficiency of TC and OTC by 5%-CN/BiP showed no significant loss, with only a slight reduction observed. This indicated the excellent stability and repeatability of 5%-CN/BiP for degradation under sunlight irradiation.

The photodegradation efficiency of 5%-CN/BiP was compared with that of other photocatalysts under visible light reported in previous literature. The detailed information is listed in Table 2. Among them, the as-prepared 5%-CN/BiP in this study demonstrated superior photocatalytic activity for TC degradation.

### 2.4. Identification of Active Species

**Figure 11:**
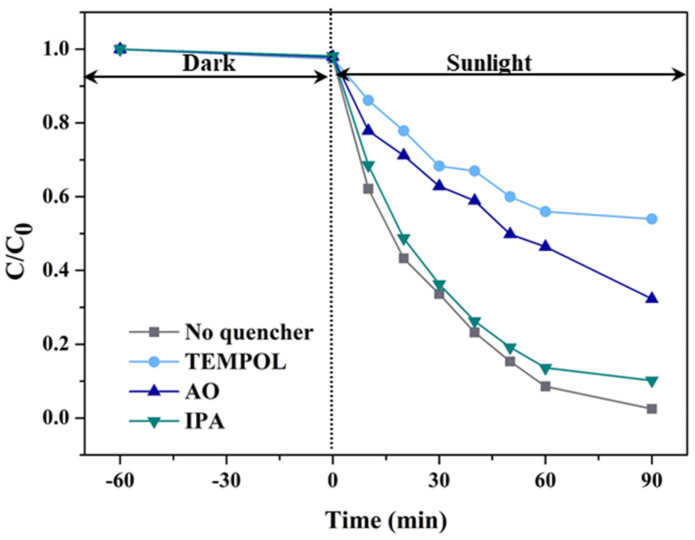
Effects of different quenchers on the photodegradation of TC by 5%-CN/BiP under sunlight irradiation.

The addition of quenching agents decreased the degradation efficiency of TC by 5%-CN/BiP (Figure 11). The performance of the samples was significantly inhibited upon the addition of TEMPOL, indicating that the •O_2_^−^ species played a dominant role in the reaction system. The introduction of AO also negatively affected the degradation rate of TC. This suggested that despite the involvement of h^+^ in the photodegradation of TC, the effect on the reaction was not as significant as that of •O_2_^−^. Conversely, the addition of IPA had a negligible effect on the degradation rate, revealing that •OH was not the primary active species.

**Figure 12:**
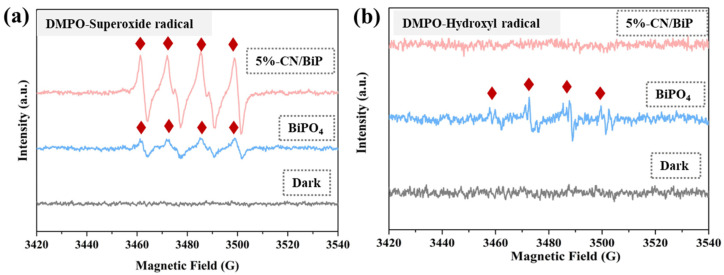
DMPO trapping EPR spectra of BiPO_4_ and 5%-CN/BiP composite in (**a**) aqueous and (**b**) methanol dispersion.

The EPR spectroscopy of BiPO_4_ and 5%-CN/BiP samples revealed that the material recombination altered the catalytic mechanism of the reaction (Figure 12). In the absence of light, no characteristic peaks were observed in all samples. However, •O_2_^−^ radicals were detected in all samples under sunlight, and the signal intensity of 5%-CN/BiP composites were stronger than that of BiPO_4_. In a single BiPO_4_ catalytic reaction system, a reduced intensity of •OH signal was obtained. Notably, almost no •OH signals were detected in the 5%-CN/BiP samples, indicating a change in the active species within the composite catalyst reaction system.

### 2.5. Mechanism of the Photocatalytic Performance Improvement

**Figure 13:**
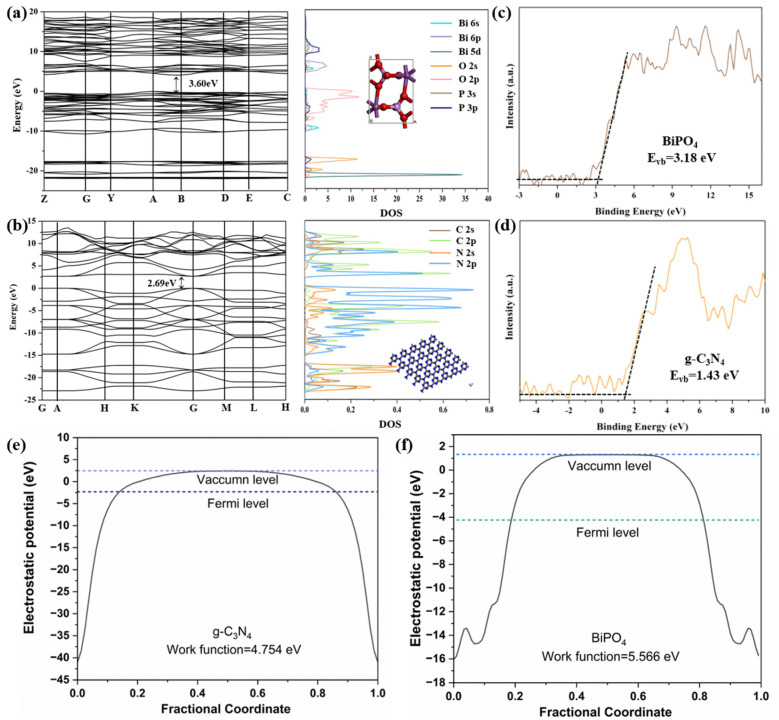
Crystal structures, calculated band structures, and density of states of (**a**) BiPO_4_ and (**b**) g-C_3_N_4_; VB values of (**c**) BiPO_4_ and (**d**) g-C_3_N_4_ calculated by XPS; Work functions of (**e**) g-C_3_N_4_ and (**f**) BiPO_4_.

The photocatalyst energy band structure and density of states calculated using density functional theory revealed the mechanism of the formed heterojunction on the catalyst photocatalytic activity enhancement (Figure 13). The results demonstrated both BiPO_4_ and g-C_3_N_4_ as n-type semiconductors with band gap widths of 3.60 eV and 2.69 eV, respectively. The band gap of g-C_3_N_4_ with amorphous structures would be lower than that for the crystalline structure [43]. The conduction band minimum of BiPO_4_ was composed of Bi 6p orbital, while its valence band maximum primarily included O 2p, Bi 6p, Bi 6s, and P 3p orbitals. For g-C_3_N_4_, the conduction band minimum mainly consisted of C 2p and N 2p orbitals, and the valence band maximum was dominated by N 2p orbitals (Figure 13a,b). The low orbital hybridization of O, P, and Bi atoms at the valence band maximum of BiPO_4_ led to a high probability of electron occurrence, whereas the orbital hybridization density of the atoms at the conduction band minimum was low. Thus, the electrons in the conduction band were easily excited to the surface of BiPO_4_. In comparison to BiPO_4_, g-C_3_N_4_ exhibited a higher degree of hybridization and electron hybridization density in the bottom atomic orbitals at the conduction band minimum, while the atomic orbitals at the valence band maximum presented a lower degree of hybridization. To confirm the electron transfer path for the built-in-electric field at the heterojunction interface, the work functions of BiPO_4_ and g-C_3_N_4_ were further calculated. As illustrated in Figure 13e,f, the work functions of BiPO_4_ and g-C_3_N_4_ were determined to be 4.378 and 4.754 eV, being in the sequence BiPO_4_ < g-C_3_N_4_. This indicated that the electrons in the g-C_3_N_4_ nanoparticles would incline to transfer to BiPO_4_. Therefore, when BiPO_4_ coupled with g-C_3_N_4_ to form an n-n heterojunction, the electrons were transferred from the conduction band of g-C_3_N_4_ with higher energy to the conduction band of BiPO_4_. Conversely, the holes in the valence band usually tended to transfer from BiPO_4_ to g-C_3_N_4_. Eventually, the formation of heterojunctions effectively suppressed the compounding of photogenerated electron–hole pairs.

**Figure 14:**
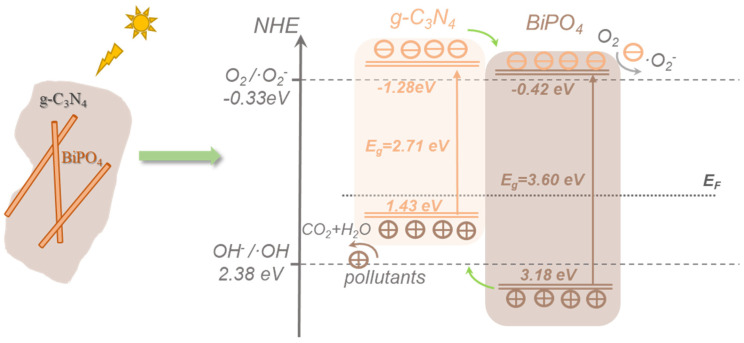
Schematic illustrations of the energy band structures upon the formation of g-C_3_N_4_ and BiPO_4_ heterojunction.

XPS analysis of the heterojunction material revealed the energy band positions of BiPO_4_ and g-C_3_N_4_. Upon sunlight absorption by the photocatalytic material, electrons in the valence band were excited to the conduction band, generating holes in the valence band (Figure 13c,d). The formation of the heterojunction contributed to the transfer of conduction band electrons from g-C_3_N_4_ to BiPO_4_, which showed a greater conduction band potential than the reduction potential of O_2_/•O_2_^−^ (−0.33 eV). Therefore, the g-C_3_N_4_/BiPO_4_ would reduce O_2_ to •O_2_^−^ [44]. Moreover, the photogenerated electrons on the surface of BiPO_4_ could trap molecular oxygen to generate •O_2_^−^, and the holes in the valence band of g-C_3_N_4_ reacted directly with the target pollutant. The valence band potential of BiPO_4_ (3.18 eV) was higher than the redox potentials of •OH/H_2_O (2.27 eV) and •OH/OH^−^ (2.38 eV) [45]. Following the addition of g-C_3_N_4_, the holes in the BiPO_4_ valence band were transferred to the valence band of g-C_3_N_4_, a valence band potential (1.65 eV) of which was lower than that of •OH/H_2_O and •OH/OH^−^. Consequently, the g-C_3_N_4_/BiPO_4_ composite could not oxidize H_2_O/OH^−^ to •OH. EPR experiments supported this conclusion: the •OH signal was detected in the reaction system using single BiPO_4_, but absent in the 5%-CN/BiP sample. Therefore, the main active species involved in the photocatalytic degradation process was probably •O_2_^−^, while h^+^ and •OH also played supplementary roles (Figure 14).

## 3. Materials and Methods

### 3.1. Materials

Tetracycline (C_22_H_24_N_2_O_8_), oxytetracycline (C_22_H_24_N_2_O_9_), tetracycline hydrochloride (C_22_H_24_N_2_O_9_·HCl), bismuth nitrate (Bi(NO_3_)_3_·5H_2_O), sodium dihydrogen phosphate (NaH_2_PO_4_·2H_2_O), urea (CH_4_N_2_O), and other chemicals used in the experiments were purchased from Nanjing Chemical Reagents Co., Ltd. (Nanjing, China) with analytically pure grade (99%), without further purification. Solutions were prepared utilizing deionized water.

### 3.2. Synthesis of BiPO_4_/g-C_3_N_4_ Heterojunction Photocatalysts

Graphitic carbon nitride (g-C_3_N_4_) was prepared using the calcination method via two stages. Specifically, 10 g urea powder was placed into an alumina crucible and calcined at 500 °C at a heating rate of 5 °C/min, followed by raising the temperature to 550 °C at 10 °C/min and maintained for 4 h. Upon cooling to room temperature, the obtained nanosheet g-C_3_N_4_ was milled into powders.

BiPO_4_ photocatalyst was synthesized using the solvothermal method. Briefly, 1 mmol Bi (NO_3_)_3_·5H_2_O and 1 mmol NaH_2_PO_4_·2H_2_O were added to 60 mL solution with deionized water and glycerol (*v*/*v*, 1:3). After vigorously stirring for 30 min, the reaction was performed in a stainless-steel autoclave at 180 °C for 24 h. Subsequently, the precipitate was collected by centrifugation and dried in an oven at 60 °C overnight.

Different mass proportions of g-C_3_N_4_ (1 wt%, 2 wt%, 5 wt%, 10 wt%, 15 wt%) were added to a mixture of 60 mL deionized water and glycerin (1:3, *v*/*v*) and dispersed evenly. Following that, 1 mmol Bi(NO_3_)_3_·5H_2_O and 1 mmol NaH_2_PO_4_·2H_2_O were continuously added and stirred vigorously for 10 min. Subsequently, the suspension was put into a stainless steel autoclave, calcined at 180 °C for 24 h, then centrifuged, collected, and dried overnight in an oven at 60 °C. The g-C_3_N_4_/BiPO_4_ composite photocatalysts with varying g-C_3_N_4_ loadings were ultimately obtained. The sample name was abbreviated as X%-CN/BiP, where X represents the mass ratio of g-C_3_N_4_, CN stands for g-C_3_N_4_, and signifies BiPO_4_.

### 3.3. Characterization of Synthetic Catalysts

X-ray diffraction (XRD) patterns of the samples were determined using an XRD-6000 X-ray powder diffractometer (Shimadzu, Kyoto, Japan) with monochromatic Cu-Kα radiation at a setting of 40 kV and 30 mA. The FT-IR spectra of the samples were determined on a Nicolet iS10 FT-IR instrument within the IR range (500–4000 cm^−1^). X-ray photoelectron spectroscopy (XPS) was performed on a PHI5000 Versa Probe spectrometer (ULVAC-PHI, Maozaki, Japan). Scanning electron microscopy (SEM) images were acquired using a QUANTA FEG 250 (FEI, Hillsboro, OR, USA), while transmission electron micrographs (TEMs) were obtained via a JEM-200CX instrument (JEOL, Kyoto, Japan).

UV–vis diffuse reflectance spectroscopy (DRS) was performed using a UV-3600 spectrophotometer (Shimadzu, Kyoto, Japan) equipped with an integrating sphere attachment, covering a wavelength range of 200–800 nm. PL were recorded on a Horiba Fluorolog 3-22 fluorescence spectrophotometer (Horiba, Irvine, CA, USA) at an excitation wavelength of 365 nm, with measurements taken over the 200–800 nm wavelength range.

Photoelectrochemical characterization was performed on a CHI760E electrochemical workstation (Shanghai, China) with a standard three-electrode system. The samples were loaded onto an ITO electrode (1 cm × 2 cm squares) and served as the working electrode. Pt plate and Ag/AgCl electrode were utilized as the counter and reference electrode, respectively. The electrolyte was 0.2 M Na_2_SO_4_ aqueous solution, and a 500 W xenon lamp (NBeT, Beijing, China) was employed to provide the light source.

### 3.4. Photocatalytic Degradation Experimental Set Up

The photocatalytic activities of as-prepared photocatalysts were evaluated by catalytic degradation of TC under visible light using 1000 W Xe lamp irradiation (Xujiang, Nanjing, China). In a typical experiment, the reactant and the catalysts were placed in a quartz tube. 20 mg of the photocatalyst was inspired into 50 mL of TC aqueous solution (20 mg/L). Prior to the light illumination, the suspension was magnetically stirred for 60 min in dark to reach an adsorption-desorption equilibrium. Following the equilibrium, the samples (volume of each is 4 mL) were taken at given time intervals, centrifuged at 7000 rpm for 10 min, and filtered through a 0.22 µm Millipore filter to remove the particles. Following that, the concentration of TC in the solution was analyzed using an Agilent 1200 high-performance liquid chromatography (HPLC) system. Ammonium oxalate (AO), isopropanol (IPA), and 4-hydroxy-2,2,6,6-tetramethylpiperidine-1-oxyl radical (TEMPOL) were added to the original reaction system as quenchers for vacancies (h^+^), hydroxyl radicals (•OH), and superoxide radicals (•O_2_^−^), respectively. The main reactive species involved in the reaction and their contributions were compared under the identical experimental conditions. Additionally, electron paramagnetic resonance (EPR) spectroscopy with 5,5-dimethyl-1-pyrroline-N-oxide (DMPO) as a trapping agent was employed to characterize radicals generated during the photocatalytic reaction. Superoxide radicals (•O_2_^−^) were detected in the methanol system, while hydroxyl radicals (•OH) were analyzed in the aqueous system. To ensure result reproducibility, duplicate experiments were performed for each condition, and average data were recorded. Blank experiments without catalysts were also performed.

### 3.5. Analytical and Computational Simulation Methods

The concentration of tetracyclines was determined using an Agilent 1200 HPLC system (Agilent, Santa Clara, CA, USA) with the UV–vis spectrophotometer 2450 and a 4.6 mm × 150 mm × 5 μm Zorbax Eclipse XDB-C18 at 35 °C. The wavelength of the ultraviolet (UV) detector was set at 355 nm. The mixture of methanol-water (10:90, *v*/*v*, 0.1% formic acid contained in water) was employed as the mobile phase with a flow rate of 1.0 ${\mathrm{mL}\;\min}^{-1}$. The photocatalytic degradation efficiency was calculated by the following Equation (4):

(4) $$\eta =\frac{C_{0}\;-\;C_{t}\;}{C_{0}}\;\times \;100\%$$

where η is the photocatalytic efficiency; C_0_ is the concentration of reactant before illumination; and C_t_ is the concentration of reactant following illumination of t hours.

The generalized gradient approximation (GGA) in the Perdew–Burke–Ernzerh (PBE) functional form was applied along with the ultrasoft pseudopotentials. Moreover, density functional (DFT) calculations were performed utilizing the plane-wave pseudopotential method in the CASTEP code [46]. The GGA with the PBE correction was also adopted to estimate the work function using CASTEP code.

## 4. Conclusions

Herein, g-C_3_N_4_/BiPO_4_ heterojunction photocatalysts were successfully prepared using the solvothermal method. The morphologies of the prepared photocatalysts were characterized, with their photoelectrochemical properties, photocatalytic activities, and photocatalytic mechanisms investigated. The results showed that the heterojunction material exhibited better photocatalytic performance compared to the single catalyst for the degradation of tetracycline antibiotics. The photocatalytic performance of the catalysts with varying g-C_3_N_4_ loadings revealed that 5 wt% of the heterojunction material exhibited the highest photodegradation efficiency for tetracyclines. The prepared heterojunction catalysts demonstrated excellent stability under sunlight irradiation, and the photocatalytic activity of the heterojunction catalysts remained almost unchanged after four cycles of recycling. Furthermore, mechanistic analysis of the photocatalytic degradation process indicated that •O_2_^−^ served as the primary active species, with h^+^ and •OH acting as supporting participants. Overall, this study offers new perspectives for the preparation of novel, environmentally friendly, and efficient visible-light-driven photocatalysts.

## Figures and Tables

**Table 1:** Photodegradation rate constants k of BiPO_4_, g-C_3_N_4_, and 5%-CN/BiP samples for TC and OTC.

Target Pollutants	g-C_3_N_4_	BiPO_4_	5%-CN/BiP
TC	7.12 × 10^−3^	7.13 × 10^−4^	3.71 × 10^−2^
OTC	7.42 × 10^−3^	1.37 × 10^−3^	2.77 × 10^−2^

**Table 2:** Summary of photodegradation efficiency of various types of photocatalysts on TC.

Type of Photocatalyst	Dosage (g/L)	C_0_ of NA (mg/L)	Light Sources	Time (min)	Removal Efficiency (%)	References
g-C_3_N_4_/BiPO_4_	1	20	λ > 420 nm	90	99.4%	This study
Ag/Ag_2_CO_3_/BiVO_4_	1	20	λ > 400	150	94.9%	[39]
Ag_2_CO_3_/Bi_4_O_5_I_2_/g-C_3_N_4_	2/3	20	λ > 400	60	82.16%	[40]
CuAl_2_O_4_/g-C_3_N_4_	0.2	100	λ > 400	60	89.6%	[41]
g-C_3_N_4_/Bi_2_O_3_@N-HMCs	2	10	λ > 420	60	90.06%	[42]

## Data Availability

Data are contained within this article.
